# Biofilm Detection by a Fiber-Tip Ball Resonator Optical Fiber Sensor

**DOI:** 10.3390/bios12070481

**Published:** 2022-06-30

**Authors:** Aida Rakhimbekova, Baizak Kudaibergenov, Damir Moldabay, Albina Zharylgap, Obinna M. Ajunwa, Enrico Marsili, Daniele Tosi

**Affiliations:** 1 School of Engineering and Digital Sciences, Nazarbayev University, Nur-Sultan 010000, Kazakhstan; aida.belova@nu.edu.kz; 2 School of Sciences and Humanities, Nazarbayev University, Nur-Sultan 010000, Kazakhstan; baizak.kudaibergenov@alumni.nu.edu.kz (B.K.); damir.moldabay@nu.edu.kz (D.M.); albina.zharylgap@nu.edu.kz (A.Z.); 3 Department of Chemical and Materials Engineering, School of Engineering and Digital Sciences, Nazarbayev University, Nur-Sultan 010000, Kazakhstan; obinna.ajunwa@nu.edu.kz (O.M.A.); enrico.marsili@nu.edu.kz (E.M.); 4 National Laboratory Astana, Laboratory of Biosensors and Bioinstruments, Nur-Sultan 010000, Kazakhstan

**Keywords:** biofilm detection, fiber-tip ball resonator, optical fiber sensor, distributed sensors, biofilm formation, biomedical sensors

## Abstract

Bacterial biofilms are one of the most important challenges that modern medicine faces due to the difficulties of diagnosis, antibiotic resistance, and protective mechanisms against aggressive environments. For these reasons, methods that ensure the inexpensive and rapid or real-time detection of biofilm formation on medical devices are needed. This study examines the possibilities of using optical- and fiber-based biosensors to detect and analyze early bacterial biofilms. In this study, the biofilm-forming model organism *Pseudomonas aeruginosa* was inoculated on the surface of the optical sensor and allowed to attach for 2 h. The biosensors were made by a fiber-tip ball resonator, fabricated through a CO_2_ laser splicer on a single-mode fiber, forming a weak reflective spectrum. An optical backscatter reflectometer was used to measure the refractive index detected by the sensors during different growth periods. The early biofilm concentration was determined by crystal violet (CV) binding assay; however, such a concentration was lower than the detection limit of this assay. This work presents a new approach of biofilm sensing in the early attachment stage with a low limit of detection up to 10^−4^ RIU (refractive index units) or 35 ± 20 × 10^3^ CFU/mL (colony formed units).

## 1. Introduction

**Figure 1:**
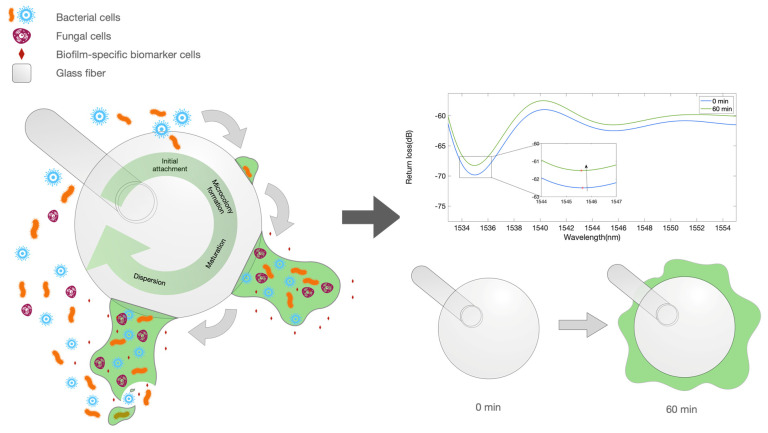
Illustration depicting the biofilm formation and biofilm markers. Left: artwork describing the process of biofilm formation on the surface of an optical fiber spherical tip used as biosensor. Right: equivalent optical model for the early stage growth, showing a ball resonator surrounded by a medium with a varying refractive index.

Currently, medicine faces a huge problem at the microscopic level—microbial biofilms [1,2]. These microstructured microbial communities are formed by both commensal and pathogenic microorganisms that colonize the epithelial or endothelial tissue of the lungs, intestines, skin, and vagina; attach to the teeth or surfaces of medical implants; or invade the host cells [3]. They can be multispecies or single species. In general, biofilm formation can be divided into five stages of development: 1—Initial reversible attachment of free-swimming (planktonic) cells; 2—Irreversible attachment of cells and microcolony formation; 3—Biofilm maturation; 4—Dispersal (Figure 1) [2,4]. In the first stage, the initial attachment of the bacteria to the surface occurs with the help of adhesion organelles of the bacteria, such as pili. This process is partially stochastic, and partly due to the response of planktonic bacteria to environmental signal (e.g., nutrient gradients and presence of a solid interface. The next stage is characterized by the production of polysaccharides and glycoproteins by bacteria to form a protective matrix termed extracellular polymeric substances (EPS) [5]. Afterwards, the biofilm grows and expands due to an increase in bacteria number and their EPS production. Inside the biofilm, bacteria can communicate using quorum sensing, that is, by transmitting chemical signaling molecules. In the final stage, the mature biofilm, in the presence of specific environmental conditions (lack of nutrients, accumulation of toxic species, etc.), partially dissolves the EPS through extracellular enzymes, thus leading to the release of planktonic cells, which restart the biofilm life cycle [4,6,7].

Gastrointestinal biofilms play an important role in human health [8]. However, most biofilms associated with human hosts enhance the infection process, favor the chronicization of the infection, and exacerbate symptoms, thus decreasing the quality of life of patients and the chances of recovery. Therefore, the detection and treatment of biofilm infections is a public health priority [9]. It is more challenging to treat or control biofilms in the host due to their resistance to antibiotics and the host immune system compared to planktonic forms of the same microorganisms. For this reason, infections caused by biofilms most often cause chronic or recurrent infections [1,2,10,11].

Healthcare-associated infections (i.e., nosocomial infections) are of particular concern, due to the antibiotic resistance of the main pathogens and because they affect individuals with decreased immune defenses. About 60–70% of nosocomial infections are implant-associated infections, which means that they are associated with some medical interventions, such as a urinary catheter (32%), surgical prosthetics (22%), artificial lung ventilation (15%), and vascular catheters (14%), etc. [3]. On average, 4–5% of all orthopedic patients who underwent surgery and 7% of all cardiac patients who received an implant suffered implant-associated infections [12].

For these reasons, it is very important to learn how to track initial biofilm formation, which enables early biofilm treatment, with a higher efficacy. Conventional methods of detecting microorganisms in patients are based on the cultivation of bacteria in vitro and finding antibodies in blood, vomit, urine, and other human body fluids [13]. Unfortunately, these methods are not effective in detecting biofilms for a number of reasons: 1—many biofilm-associated microorganisms are difficult to cultivate or uncultivated species; 2—nosocomial biofilms comprise multiple species, which demands a species-independent method for biofilm detection, rather than species-specific approaches based on DNA fragments and antibodies; 3—there are substantial differences between biofilms grown in vivo and in vitro; 4—EPS allows bacteria inside the biofilms to hide from antibodies, immune cells and even bacteriophages; 5—biofilm biomarkers are very small in size and concentration, which requires more local measurements [7]. Current techniques for the identification and characterization of biofilms are based on offline imaging (e.g., confocal laser scanning microscopy), which is expensive and requires expert operator [7]. Further, microscopy is not a suitable technique for in vivo examination, excluding biofilms that have infected exposed surfaces, such as skin and mucous membranes.

Therefore, there is a need to detect the formation of medical biofilms in real-time, providing a quantitative output. At the same time, the methods to implement such detection should be inexpensive, compact, biocompatible, and easy to fabricate for high-volume manufacturing (i.e., production of disposable sterile devices). These sensors should serve preventive epidemiological purposes, that is, prevent the contamination of the host by changing the catheter/drainage in time.

For these purposes, one interesting approach is the use of optical fiber sensors as a tool for inspecting biofilm growth. Currently, optical devices are widely used in microbiology to count the number of bacteria but not to determine biofilms [14,15]. However, some studies are already being conducted to examine biofilms using optical fiber biosensors (OFBs). According to recent studies, optical sensors are able to detect the growth of biofilm mass on their surface (Table 1) [16,17]. The basic principle of operation of OFBs is the detection of changes in the refractive index (RI) of the medium surrounding the sensing element. These biosensors can either work in the so-called “volume RI” condition, where the sensing element is placed in a homogeneous medium with varying RI, or in the “surface RI” condition, when the immobilization of elements with much smaller dimensions and skin depth than the wavelength elements (e.g., proteins) occurs on the surface of the sensor [18,19].

OFBs have strategic advantages both in terms of form factor and performances over other electrochemical or optical methods [20]: they have a compact size and lightweight structure, with a thickness of less than one millimeter; they are biocompatible in accordance with ISO 10,993 standard; they are inexpensive as they heavily rely on telecom standards, particularly when using single-mode fibers (SMFs) at infrared wavelengths around 1550 nm; they enable real-time detection with an interrogation unit. The main results relating to the use of OFBs for monitoring the biofilm formation are reported in Table 1. Kurmoo et al. [16] reported a long-period grating (LPG) biosensor for the detection of *Pseudomonas aeruginosa* biofilm formation, achieving a repeatable exponential correlation between optical signal and biomass. Yuan et al. [17] demonstrated the use of a tilted fiber Bragg grating (TFBG) for measuring extracellular electron transfer in electroactive biofilms (EABs), which allows for a more in-depth study of electrochemical processes inside biofilms.

In this study, we report the use of a fiber-tip ball resonator (BR) fiber-optic sensor for monitoring the growth of *P. aeruginosa* biofilm in the early stage. In comparison to LPG and TFBG sensors, BR sensors have additional properties that make them more suitable for this application. First, BR sensors are much faster and cheaper than any grating, since they can be fabricated using CO_2_ laser splicer adapting methods used in whispering gallery mode resonators [21,22,23] or the microbubble resonators [24] manufacturing of single-mode telecom fibers [25]. In addition, BR sensors work as reflective units, with a quasi-random spectrum formed by shallow polarization-sensitive fringes that resemble a Fresnel probe, and can be interrogated either by measuring the intensity change or the wavelength shift of a spectral peak [25,26]. Due to a very low reflectivity, the interrogation of a BR sensor can be performed with an optical backscatter reflectometer (OBR). Another advantage of BR sensors, as well as for optical fiber sensors with refractive index measurement capability, is the possibility of multiplexing, by simultaneously scanning multiple sensing units separated by time/wavelength (such as for gratings), or in spatial division multiplexing networks, which are particularly effective when operating with broadband spectral sensors [27].

The illustration in Figure 1 displays the working principle of the BR sensor in measuring the bacterial growth. The left artwork shows the process of biofilm growth, which occurs around the whole fiber tip. The growth of this film around the fiber device, in the early stage, forms a layer around the surface that is interpreted as a change in the refractive index (RI), causing the reflection spectrum to change. This effect appears to saturate when the sensor is completely surrounded by a thick film; however, the high sensitivity of the BR sensing units allows for a detection of the early part of the biofilm formation process.

## 2. Materials and Methods

### 2.1. Materials

Pure cultures of *P. aeruginosa* ATCC 10145 were supplied by Microbiologics (St. Cloud, MN, USA). Nutrient agar, nutrient broth and parafilm^®^ were obtained from Sigma-Aldrich (St Louis, MO, USA). Centrifuge tubes of 2, 15 and 50 mL were supplied by Corning^®^. Intravenous catheters with injection valve of size G18 were used from trademark “IGAR”.

### 2.2. Fiber Calibration

**Figure 2:**
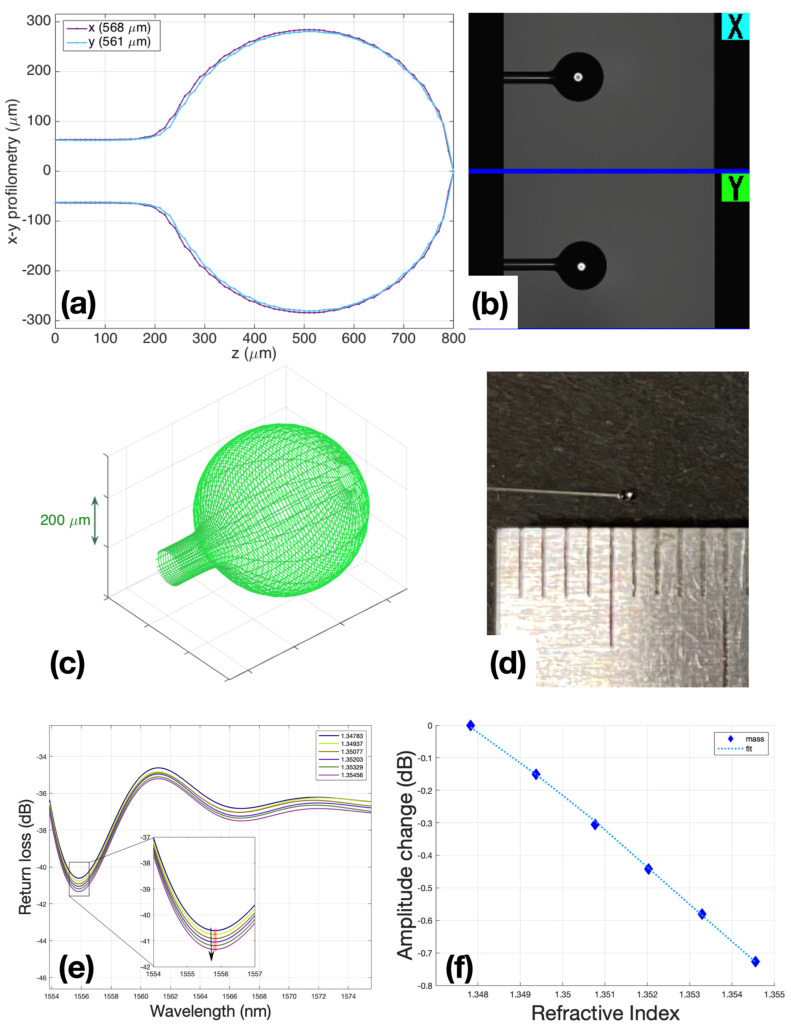
Ball resonators fabrication: (**a**) BRs profilometry; (**b**) microscopic view of BR in the Fujikura’s interaction window with the operator; (**c**) BRs 3D modeling view; (**d**) BR view with 500 um diameter; (**e**) spectral change in BR during calibration with 200 μL steps of sucrose 40, while the inset shows the spectral feature detected by intensity measurement; (**f**) fitting of calibration R^2^ = 0.9997, and sensitivity is −108.38 dB/RIU.

Fabrication of ball resonators (BRs) or fiber-tip spherical resonators were derived from the fabrication of the whispering gallery mode (WGM). Ball resonator sensors were used to detect biofilm formation. BR resonators used as biosensors in this study are simple and highly sensitive refractive index (RI) sensors [26,28]. For this study, ball resonators with a diameter of about 500 μm were manufactured from commercial SMF fibers (Corning SMF-28), using the process reported in Figure 2, and interrogated using telecom-grade equipment operating in the third optical window.

The principle of operation of the ball resonators is based on the effect of interferometry with a shallow fringe and a spectrum [26,28,29]. In other words, a change in the intensity and a slight wavelength shift occur when the refractive index of the environment or the sensor surface changes. Since the output signals were very small and chaotic, it was necessary to use a sensitive reflectometer to determine RI change and differentiate it from side noise.

Ball resonators were made from simple single-mode fibers using a special Fujikura LZM100 (Fujukura, Japan) fiber splicer device. This device produces a spherical sensor by heating two optical fiber segments with a CO_2_ laser. Currently, the CO_2_ laser is widely used in the manufacturing of optical fibers of various shapes and applications. It heats the surface of the glass to melt it and achieve a desired shape without unnecessary damage to the surface, debris particles, and chemical elements. Briefly, after splicing two equal cut ends of fibers, the fibers were pushed from opposite ends under the hit, and the device began a fast rotation that formed a ball near where the fiber was spliced and broken. 

After the sensors were manufactured, they were calibrated to make sure that the sensors directly responded to changes in the refractive index of their environment (air, liquid). Calibration was carried out according to the standard method: using a refractometer Luna OBR 4600, RI was measured in 6 ml of 10% sucrose solution, then 200 μL of 40% sucrose was added in each subsequent step, bringing the amount to 1 mL. A total of 6 measurement points were used to post the graph points and check the linearity of the sensor response. Only sensors with R^2^ (Coefficient of determination) ≥ 0.95 were used for experiments (Figure 2f).

The performances of the sensors used in all experiments are reported in Supplementary Material Table S1; the BR sensitivity ranges from −90.59 to −144.28 dB/RIU, while the minimum return loss obtained in the spectral dips where the detection occurred was around ~−70 dB. Conversely, an ideal Fresnel probe [30] shows a spectrally flat return loss of −27 dB, and sensitivity of −54 dB/RIU for values of a refractive index similar to those reported in this study.

### 2.3. Experimental Setup

**Figure 3:**
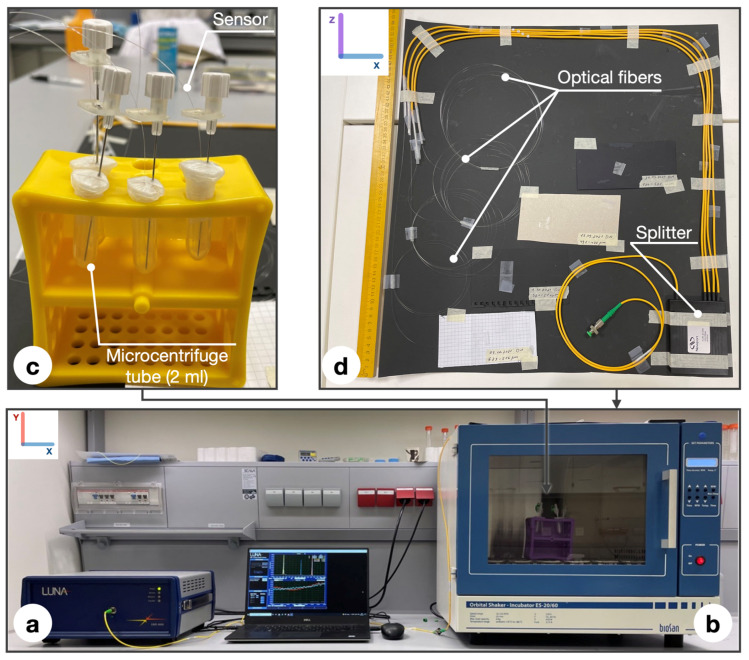
Set up of the experiment: (**a**) OBR Luna 4600 with a computer used for data acquisition; (**b**) incubator; (**c**) rack with probes consisting of microcentrifuge tubes (2 mL), parafilm wrapping, intravenous catheters (G18) containing ball resonator; (**d**) multiplexing set up consisting of splitter and optical fibers.

The experimental setup of the biofilm detection procedure shown in Figure 3 comprises the following parts: (a) Optical backscatter reflectometer (OBR, Luna 4600, Roanoke, VA, USA) with a computer used to collect and process the data; (b) incubator; (c) rack with probes; (d) multiplexing set-up.

The working principle of the ball resonator sensor, applied to refractive index sensing [26,28], could be extended in this study, as shown in Figure 1. As the bacterial layer grows on the surface of the sensor, it forms a layer between the fiber surface and the outer layer, which causes an increase in the surrounding refractive index (similar to the volumetric RI measurements carried out with a BR probe reported in [28]), which in turn results in a change in the intensity recorded by the OBR. This phenomenon is particularly visible in the early stage of the biofilm formation, and appears to saturate when the ball on the fiber tip is covered by a thick layer, as depicted in Figure 1.

To measure biofilm formation, a multiplexing design consisting of 3 simultaneously working sensors was used. Multiplexing set up was placed on the roof of the incubator because it has a large area. A large area was used to avoid return losses due to a twisting of the fiber that was too tight. The photo of the device shows that, for the stable position of the resonators and the exclusion of changes in amplitude by tension, we decided to use intravenous catheters of size G18. All sensors were pretreated and placed in sterile 2 mL tubes and sealed with parafilm to ensure the anaerobic conditions of the system. The essence of the multiplexing set-up is the simultaneous measurement of 3 sensors, which is a great advantage for measuring complex biofilms [27]. Additionally, during data analyses, the fact that all 3 measurements were carried out under the same conditions, makes them more reliable. In order to distinguish each sensor during measurements on a refractometer in a multiplexing set-up, it is necessary to divide the sensors into adequate distances, so that there is at least 1 m of distance between them: the 1st resonator is located at 1 m on the splitter, the 2nd at 2 m, and the last at 3 m, respectively. After all measurements, data were analyzed using Matlab software. Limit of detection calculation is described in the Section 4.

### 2.4. Microbiological Methodology

**Figure 4:**
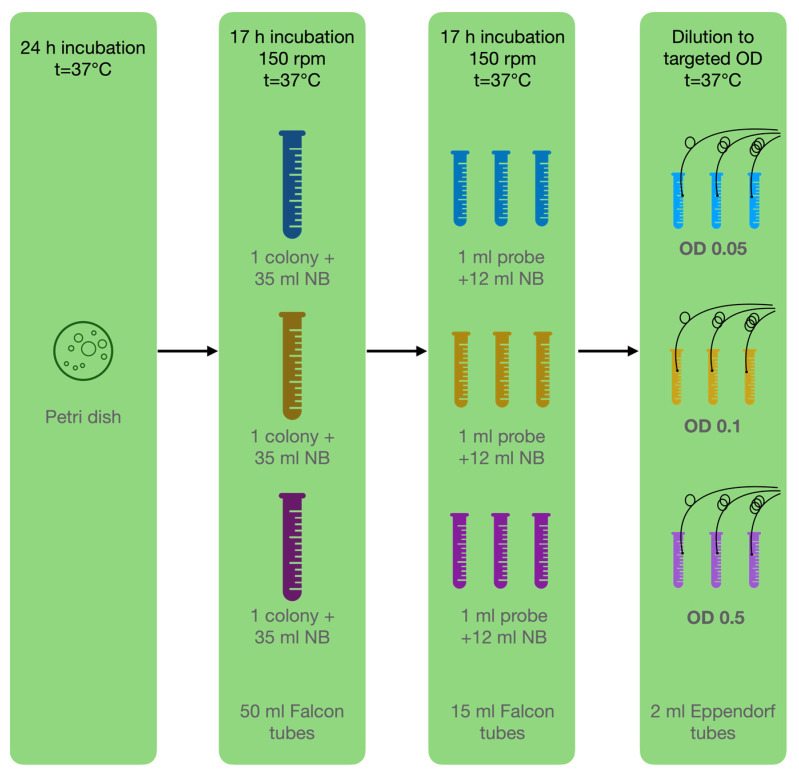
Microbiological methodology: Step 1—cultivate bacteria on Petri dish; step 2—inoculation of one pure colony in 3 tubes with NB; step 3—inoculation of 3 separate replicants for each experiment in NB; step 4—dilution to targeted OD for experiment.

In this study, bacteria attached to the surface of the ball resonator and caused a shift in the refractive index of the environment. For the experiments, a pure culture of *P. aeruginosa* ATCC 10,145 was used and cultivated following the basic protocol described by LaBauve and Wargo [31]. The bacterium was cultivated as colonies on nutrient agar in Petri dishes for 24 h, and then subcultured to a liquid medium for 17 h of incubation at 37 °C. Prior to the experiment, the cultures were diluted to the target optical density (OD): 0.05, 0.1, 0.5; using sterile nutrient broth warmed to 37 °C (liquid medium used for the cultivation of a wide variety of microorganism, suitable for microbiology, NutriSelect^®^ Plus) to eliminate the effect of temperature differences, which also affect the optical sensors (Figure 4). All the test tubes were placed in an incubator in static mode at a constant temperature of 37 °C to maintain the optimal growth of *P. aeruginosa*.

Following the first hour, the sensor no longer responded to environmental changes. For this reason, we decided to measure different concentrations for 2 h with an interval every 2 min. The number of attached cells was independently measured through a crystal violet binding assay after 1 and 2 h of incubation [32]. Firstly, the planktonic cells were removed, and the BR was washed 3 times in sterile water by gentle immersion for one second and allowed to dry for 3 min. Secondly, the cells were fixated by immersion in 99% methanol for 3 min, followed by 1 min drying. Thirdly, 2 mL of crystal violet solution (0.2%) was added to all wells. After 15 min, the excess crystal violet was removed, and the plates were washed twice and dried in air. Finally, the crystal violet bound to the biofilm mass was dissolved in 33% acetic acid. The attachment of bacteria to form a biofilm was determined by measuring the OD of the dissolved biofilm mass in 600 nm acetic acid using a multimodal spectrophotometric reader (Thermo Scientific Varioskan LUX, Waltham, MA, USA).

## 3. Experimental Results

**Figure 5:**
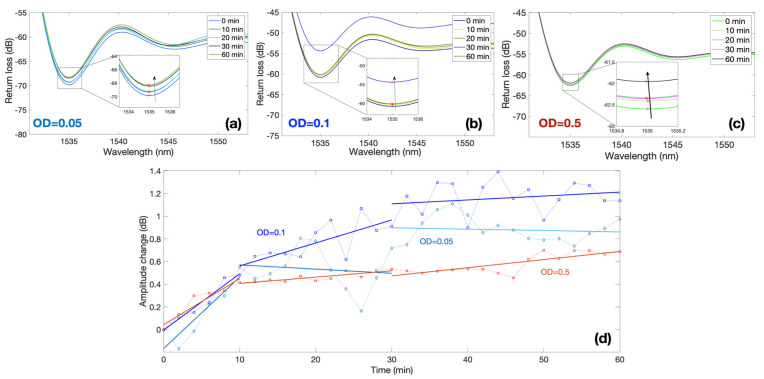
Spectral response of ball resonators in different bacteria concentrations; the charts report the response of three different sensors with sensitivity values −127.52, −144.28 and −104.46 dB/RIU at different values of OD recorded over 60 min period. (**a**–**c**) Reflection spectra of each sensor for different measurement times for OD equal to 0.05 (**a**), 0.1 (**b**), and 0.5 (**c**). Insets of each figure show the spectral valley used for the intensity level tracking. (**d**) Normalized response for each sensor, reporting the amplitude change over time; the response of each OD is separated into 3 regions: 0–10 min, 10–30 min, 30–60 min.

Measurements were performed focusing on the first 2 h and sampling the response of each sensor every 2 min. Figure 5 shows the response of three BR sensors, for bacteria with an optical density (OD) ranging from 0.05 to 0.5, and focuses on the first 60 min of the experiment, where the sensors show a clear response.

The first three results show the spectra of each BR sensor and the spectral feature used for the interrogation. Each sensor has a different spectrum, which appears as an almost random pattern, as previously displayed in [26,28]. The first sensor with a sensitivity −127.52 dB/RIU displays a value of around 1535 nm; by recording the spectrum at this wavelength value, we observe a progressive increase in intensity from 0 to 60 min, equal to approximately 1 dB. A similar trend is observed for the second sensor (−144.28 dB/RIU) exposed to OD = 0.1 and for the third sensor (−104.46 dB/RIU) immersed in suspension with OD = 0.5.

In order to compare measurements of multiple sensors, each with a different sensitivity, Figure 5d shows the normalized output of each sensor obtained by dividing the intensity change by the sensitivity, therefore obtaining the equivalent RI change. This is reported in the chart for all the three values of ODs, highlighting the time response ranging from 0 to 1.2 dB. The graph shows that the amplitude change is greatest in the first 10 min in samples, regardless of the initial concentration of bacteria. At this time, the sensor shows the maximum response: approximately 0.5 dB in 10 min. The next shift of amplitude by 0.5 dB in response to environmental changes (attachment and colonization of bacteria) already takes 20 min (from 10 to 30 min). In the second half of monitoring (from 30 to 60 min), the amplitude shift becomes stable.

**Figure 6:**
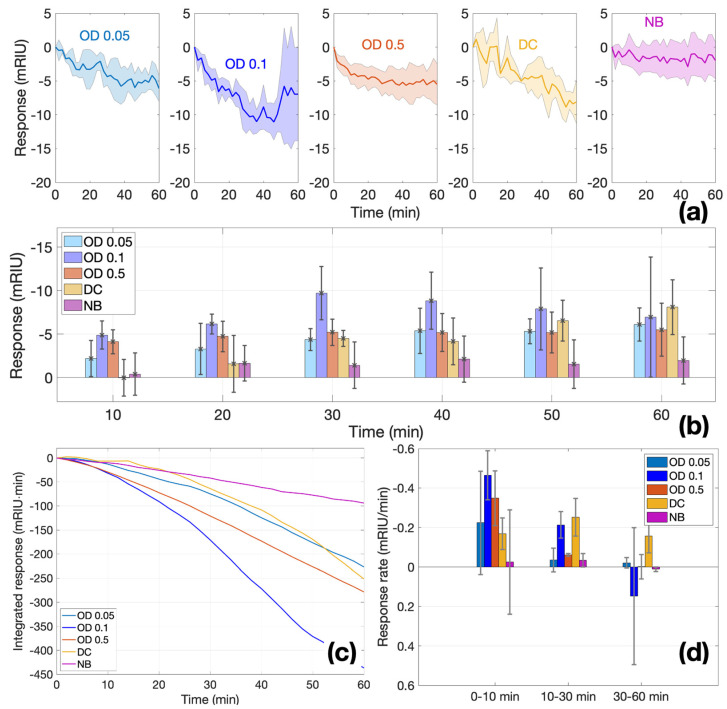
Time response of ball resonators in different bacteria concentrations. (**a**) Normalized response during the 0–60 min time interval for each value of OD (0.05, 0.1, 0.5) and controls (DC, NB); charts report the average (solid line) and ±standard deviation (shadowed region) of 3 different sensors. (**b**) Bar chart reporting the temporal response in each condition, at each time stamp (bar = mean; error bar = ±standard deviation). (**c**) Integrated response, showing the integral of the average response at each time. (**d**) Response rate, estimating the slope of each normalized response over three time intervals (0–10, 10–30, 30–60 min); bar = mean; error bar = ±standard deviation.

As a result of measurements, trends (Figure 6d) of the sensory response were revealed in the first hour; after this time, the sensor stopped responding to changes in the biofilm mass. Different sensors were used for each experiment and normalized to a common assessment scale. The measurements were carried out by completely independent sensors at the same time. Figure 6a clearly shows that the sensors react by lowering the amplitude shift in the first hour of monitoring, and this trend continues throughout the entire monitoring time. By doubling the concentration of bacteria in the nutrient broth (NB) in the first hour, a maximum is reached on the sensor surface at which the sensor can respond to changes. After that, the sensor does not respond to an additional increase in the amount of bacteria attached to the surface. At a maximum concentration of OD = 0.5, this response is smoother and reaches its maximum per hour, after which the graphics even aligns upwards, which may also be explained by a change in the surface charge of the sensor due to bacteria. For the control group, two experiments were conducted: using sterile nutrient broth without the addition of bacteria and sterile nutrient broth (NB) with bacteria killed in an autoclave, which should not show their ability to attach to the surface. The graph of the nutrient broth (NB) control (Figure 6a) shows no changes from the sensor during the entire monitoring time, while with the dead cells (DC), a strong sensor response is visible in the form of an amplitude shift of +1.2 mRIU. Reasons for the response elicited by the DS setup are explained in the Section 4.

A complete analysis of all experiments, including the replicates, is shown in Figure 6. Figure 6a shows the average and standard deviation of the response for each measurement condition, and in Figure 6b, the responses sampled for each 10 min time period are displayed. In Figure 6b, at both the lowest concentration (OD = 0.05) and twice the level of concentration (OD = 0.1), the sensor exponentially increases its response in the first 30 min, after which the sensitivity drops. At the same time, at the highest concentration (OD = 0.5), already in the second 10 min period, they reach a mass at which the sensor can no longer detect changes on the surface. The control group with no cells (NB) over time remains relatively stable at the level of 1–2 mRIU. Although the control group with dead cells (DC) shows a gradual growth within an hour, such linear growth does not correspond to the vital activity of bacteria and most likely means that bacteria settle on the sensor surface over time.

In the integral analysis of all experiments (Figure 6c), we can see a rapid response of the sensor to the attachment of cells to the surface of the glass in the first 30 min, after which the response becomes relatively stable. In samples with a higher concentration (OD = 0.5), where there are more bacteria and therefore more probabilities of attachment, the sensor response stabilizes faster than at lower concentrations, such as OD = 0.1 and OD = 0.05. A more detailed analysis of the rate of a ball resonator response shows that the maximum efficiency of the sensor in detecting bacterial attachments is manifested in the first 10 min, while after 30 min, the sensitivity begins to decrease because the layer of attached bacteria exceeds the detection zone of changes on the sensor surface (Figure 6d). This trend may indicate the detection of changes on the surface of the ball resonator glass, more specifically, the determination of the bacterial attachment (the first stage of biofilm formation).

**Figure 7:**
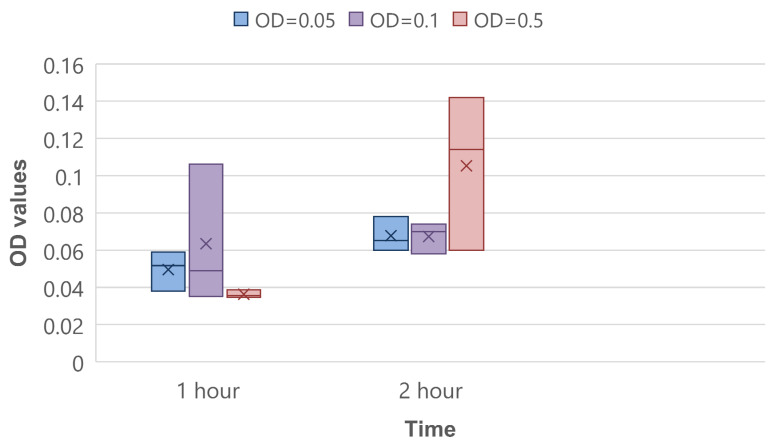
CV binding assay for 1 and 2 h of bacteria incubation.

The CV binding assay was performed as quantitative control of the biofilm’s initial attachment stage. On average, there was a shift from 1 h to 2 h of incubation on 0.018, 0.004, and 0.069 optical density units in experiments of OD = 0.05, OD = 0.1 and OD = 0.5, respectively (Figure 7). This proves that the amount of bacteria on the sensor surface increases.

## 4. Discussion

In this paper, we proposed using ball resonators as an alternative to detect biofilms. If we compare ball resonators with sensors used in previous similar works (TFBG and LPG), the ball resonator proves to be a more sensitive and cheaper biosensor for determining the attachment of bacteria. It is also a huge advantage that a multiplexing setup can be used, since cells attachment and biofilm formation are poorly reproducible processes.

Conventionally, bacterial biofilms can be detected by various methods. A culture plate technique is considered a gold standard method, where bacteria are placed in multiple-well plates. Each plate is filled with 0.1–0.2 mL of broth, either containing a culture of required bacteria or a negative control without bacteria. Then, the plates are incubated at the required temperature for specific bacterial growth for 18–24 h, washed with phosphate-buffered saline (PBS), and the remaining biofilms on the walls of the plate are fixed with crystal violet stain. Finally, plates are tested for optical density on an absorbance reader. Plates with the remaining violet stain are considered to have a formed biofilm [3,33].

The next method is tube adherence, where a test tube containing broth is inoculated with bacteria, incubated at 37 °C, stained with crystal violet, and washed with PBS and water. During the test, tubes are placed in an inverted position and are then observed for staining with CV on the walls and the bottom of the tube. This is a qualitative test, where a score of 1 suggests weak to none, 2 suggests average, and 3 suggests strong biofilm formation [33,34].

Another method is Congo Red Agar, in which a Congo Red stain is added to sterile agar broth, followed by inoculation with bacteria and 24 h incubation at 37 °C in an aerobic environment. The appearance of black-colored colonies is considered a positive result of biofilm production, in contrast to red colonies, which suggest that the biofilm was not formed [12].

All these methods proved to be reliable and convenient methods to discover newly formed biofilms. However, all of them require at least 18 h incubation, which means that these methods are not capable of quick detection, and thus are not suitable for the real-time tracking of biofilm growth from the earliest stage—cell attachment. Comparatively, optical fiber sensors have a very high precision as they are able to detect the smallest changes on the surface of an optical fiber sensor [16,17]. In addition to a high precision, the sensor itself is a very thin device, which can be inserted within a medical catheter, blood vessel, and other inaccessible places, which can often become contaminated by pathogenic microorganisms.

The results turned out to be more chaotic than expected, but the reasons for this may be down to the variability of the microbiological part of the experiment, as well as the inability to create identical biofilm replicates. From a microbiological point of view, further research on measurements with different bacteria and growth conditions to determinate reproducibility are needed to statistically confirm that the measurements can be considered identical.

There is some difficulty in determining the exact number of bacteria attached to the sensor as there is no existing model for quantitatively correlating the attachment of bacteria to the sensor as the sensor approaches saturation. As the sensors become saturated, this is the point where detection stops. From the point of the first attachment of cells to the point of saturation, it is difficult to find the minimum number of bacteria detected by the optical sensor (limit of detection). This is combined with the fact that during such a transition from the initial attachment to the saturation point of the sensor, there may be other hypothetical biophysical factors at play that can affect attachment, such as: fluid flow dynamics, surface tension of the medium and surface chemistry and charge of the bacteria. Additionally, the method of detecting the amount of biofilm by the crystal violet binding assay method is less effective for the following reasons: a low reproducibility of results [35,36] uneven staining and differential removal of biofilm, as well as non-selective binding of the dye [37].

Essentially, this technique targets cell biomass attachment and not active metabolic activity of the cells. Therefore, the values detected in the dead cells (DC) set-up could be caused by the settling of dead biomass on the sensor surface based on simple particulate interactions between the sensor and dead cells. This accounts for the values obtained in the DC experiments in this study. For the live bacterial cells analyzed in this study, a general trend in all samples was observed. For the first hour, the sensor shows an amplitude shift of 5–7 mRIU. However, we observed that a saturation point was reached, and another important factor that affects sensor response is the location where the bacteria are initially attached. The sensor response is greater if cell attachment and changes occur at the top of the sensor. Additionally, the transition from a planktonic form to a biofilm is more probable in environments with a low nutrient content and during flow. The initial attachment process only starts after a specific environmental signal tern on the genetic program in planktonic cells [38]; then, these bacteria swim in all directions to and from the sensor, which makes this process non-linear. The fact that dead cells show binding to the sensor surface can possibly be explained by the retained ability of lysed cells to stick to the glass surface in sufficient quantity to evoke the sensor response.

The limit of detection was calculated using the fundamentals of mathematics and an understanding of optical sensing. Since the Luna refractometer’s resolution is 0.01 dB and the ball resonators’ sensitivity is on average 100 dB/RIU, the optical RIU accuracy is evaluated by Equation (1):

0.01 dB/100 dB/RIU = 10^−4^ RIU. (1)

According to Figure 6d, sensors respond from 0.22 to 0.46 mRIU per minute, which is on average 0.34 × 10^−3^ RIU/min. Equation (2) shows that the optical accuracy is divided by the average sensor response over time, and the time taken for bacterial attachment mass to induce the sensors’ amplitude shift is calculated:

10^−4^ RIU/0.34 × 10^−3^ RIU/min = 0.3 min. (2)

If we assume that the limit of detection is equal to how many bacteria attach in 0.3 min, we can convert it to CFU/mL. In Figure 7, the OD changes in attached bacteria in 1 h are presented. It follows that the OD on average changes by 3 × 10^−2^ in one hour. Equation (3) presents the OD change over 0.3 min:

3 × 10^−2^ × 0.3 min/60 min = 1.5 × 10^−4^. (3)

The value of OD change per 0.3 min with standard deviation and error is 17 ± 9.9 × 10^−5^. Dong-ju Kim et al. determined that 1 OD = 2.04 × 10^8^ CFU/mL, which allows us to convert the limit of detection of the ball resonator to the equivalent number of attached cells’ biomass, according to Equation (4) [39].

17 ± 9.9 × 10^−5^ × 2.04 × 10^8^ CFU/mL = 35 ± 20 × 10^3^ CFU/mL. (4)

These calculations are an approximate comparison of the ball resonator’s limit of detection aligned in terms of biomass concentration.

The technique of using optical fibers to determine biofilm attachment is still in its formative and most challenging stages. Currently, not enough research focuses on this area. There is still a gap in the utilization of this technique as it has not been made functional for the specific determination of cellular structures. Further experiments are required to develop the technique and utilize it in the proper evaluation of cellular activity, and not just cell attachment.

## 5. Conclusions

Ball resonators are highly sensitive sensors for detecting changes on the surface, such as the primary attachment of the cell. Its sensitivity is high in a low concentration of bacteria, but it decreases in concentrated media, which is explained by the low diameter of the surface detection zone. It means that the sensor does not respond to surface changes after a certain number of bacteria attach to it. The low diameter of the surface detection zone can only accommodate a small number of cells that essentially translate into the initial early attachment of cells in biofilms.

Another conclusion that can be made is that the sensor’s high sensitivity to the early primary attachment of bacteria could serve as an excellent indicator in the medical field for monitoring the sterility of devices.

In conclusion, optical fiber biosensors based on ball resonators provide an affordable, rapid, and multiplexed method to measure biofilm growth over the surface of a sensing unit. In this study, the experimental results show a detectable change in intensity, occurring for each sensor, on the order of >0.4 dB over the first 10 min, and then a stable level over ~60 min is achieved when the sensor is completely coated. The results of this study show the potential for a real-time monitoring of the early stage of biofilm formation, which complements other electrochemical or optical methods that are more suited for long-term measurements.

Future research will revolve around improving the specificity of detection, designing sensors that can better differentiate between the OD of the biofilm and the surrounding medium, as well as incorporating this method into a sensing system that can track both the early and late stages of the biofilm formation process.

## Figures and Tables

**Table 1:** Main results obtained with optical fiber biosensors in monitoring biofilm formation; the table shows the OFB methods, types of biofilm, sensitivity, limit of detection (LoD), and technology readiness level (TRL).

OFB	Coating	Analyte	Sensitivity	Method	LoD	Bacteria	Reference
LPG	None	Biomass of *P. aeruginosa* biofilms grown	0.002 nm/μg cm	SMA-optical spectrometer	81 μg cm^−2^	*P.aeruginosa* biofilm	[16]
TFBG	Gold	Electrochemical changes	0.01 nm/RIU	EC-SPR-OFS	2.6 μA/cm^2^	*G. sulfurreducens, Sh. oneidensis, E. coli* biofilm	[17]
Ball resonators	None	Biomass of *P. aeruginosa* biofilms grown	91–170 dB/RIU	SMF-optical reflectometer	~10^−4^ RIU	*P. aeruginosa* biofilm	This study

## Data Availability

Data presented in this work are not publicly available at this time but can be obtained upon reasonable request from the authors.

## Supplementary Materials

The following supporting information can be downloaded at: https://www.mdpi.com/article/10.3390/bios12070481/s1, Figure S1. Ball resonator fabrication by CO_2_ laser. Figure S2. Settings of ball resonators manufacture: (a) Physical characteristics settings; (b) Heating settings; (c) Rotation settings; (d) Movement settings; (e) Measurements of fabricated ball resonators. Figure S3. View of all response of all experiments over time. Figure S4. Scanning electron microscopy of OD = 0.5 sample. The yields of initial attachments of bacteria are seen. Figure S5. Spectra of ball resonators: (a–c) ball resonators used for OD = 0.05 experiment with diameters 535–541 μm, 541–534 μm, 526–521 μm, respectively; (d–f) ball resonators used for OD = 0.1 experiment with diameters 569–561 μm, 544–538 μm, 543–539 μm, respectively; (g–i) ball resonators used for OD = 0.05 experiment with diameters 532–523 μm, 523–521 μm, 520–513 μm, respectively. Figure 6. Spectra of ball resonators used for controls: (a–c) ball resonators used for control DC with diameters 565–559 μm, 567–564 μm, 571–564 μm, respectively; (d–f) ball resonators used for control NB with diameters 568–561 μm, 569–563 μm, 570–564 μm, respectively. Table S1. Size and sensitivity of all BRs used in experiments (NB—nutrient broth, DC—dead cells, bacteria OD = 0.5, OD = 0.1, OD = 0.05).
