# Supplementary material for: Biofilm Detection by a Fiber-Tip Ball Resonator Optical Fiber Sensor
Source: Biosensors (Basel). 2022 Jun 30;12(7):481. doi: 10.3390/bios12070481 (PMC9313161; doi:10.3390/bios12070481)
Supplement: Supplementary file 1 [file biosensors-12-00481-s001.zip › biosensors-1781154-supplementary.pdf]

Biofilm Detection by a Fiber-Tip Ball Resonator Optical Fiber Sensor

Aida Rakhimbekova [1], Baizak Kudaibergenov [2], Damir Moldabay [2], Albina Zharylgap [2], Obinna M. Ajunwa [3], Enrico Marsili [3] and Daniele Tosi [1,4,*]

[1] School of Engineering and Digital Sciences, Nazarbayev University, Nur-Sultan 010000, Kazakhstan; aida.belova@nu.edu.kz
[2] School of Sciences and Humanities, Nazarbayev University, Nur-Sultan 010000, Kazakhstan; baizak.kudaibergenov@alumni.nu.edu.kz (B.K.); damir.moldabay@nu.edu.kz (D.M.); albina.zharylgap@nu.edu.kz (A.Z.)
[3] Department of Chemical and Materials Engineering, School of Engineering and Digital Sciences, Nazarbayev University, 53 Kabanbay Batyr Ave., Nur-Sultan 010000, Kazakhstan; obinna.ajunwa@nu.edu.kz (O.M.A.); enrico.marsili@nu.edu.kz (E.M.)
[4] National Laboratory Astana, Laboratory of Biosensors and Bioinstruments, Nur-Sultan 010000, Kazakhstan
* Correspondence: daniele.tosi@nu.edu.kz

Supplementary Materials

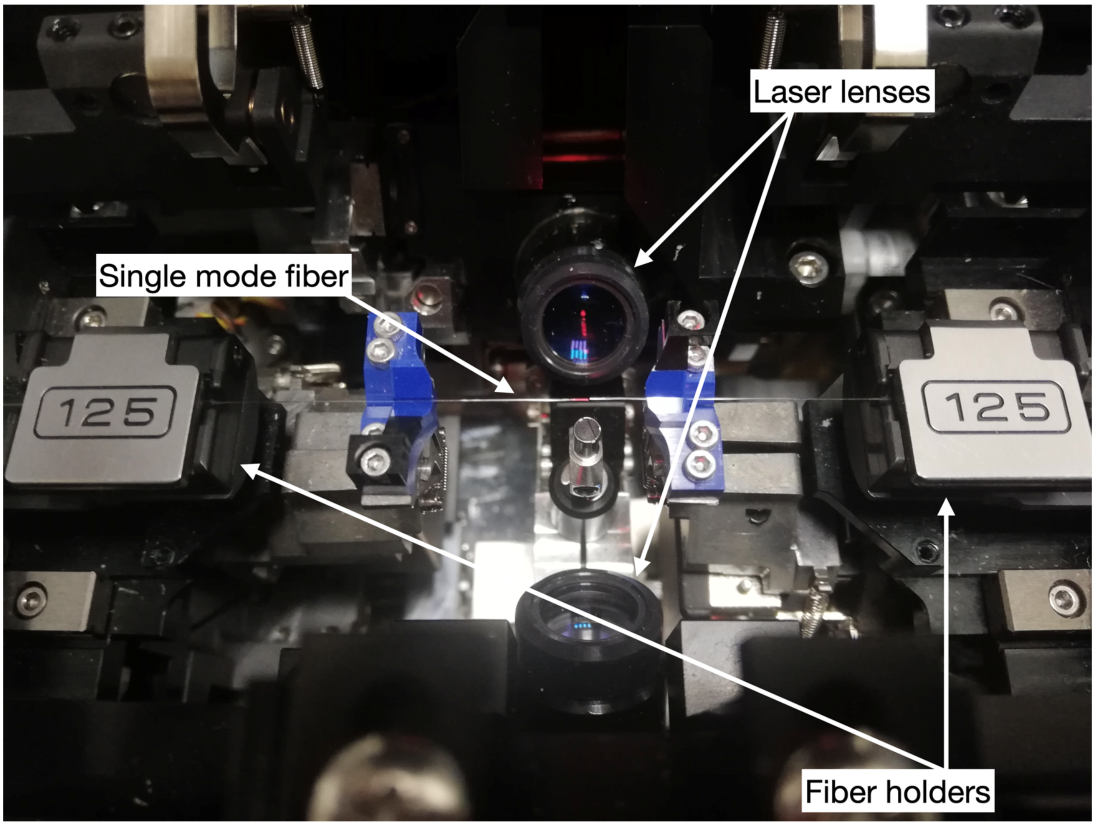

Figure S1. Ball resonarot fabrication by CO2 laser.

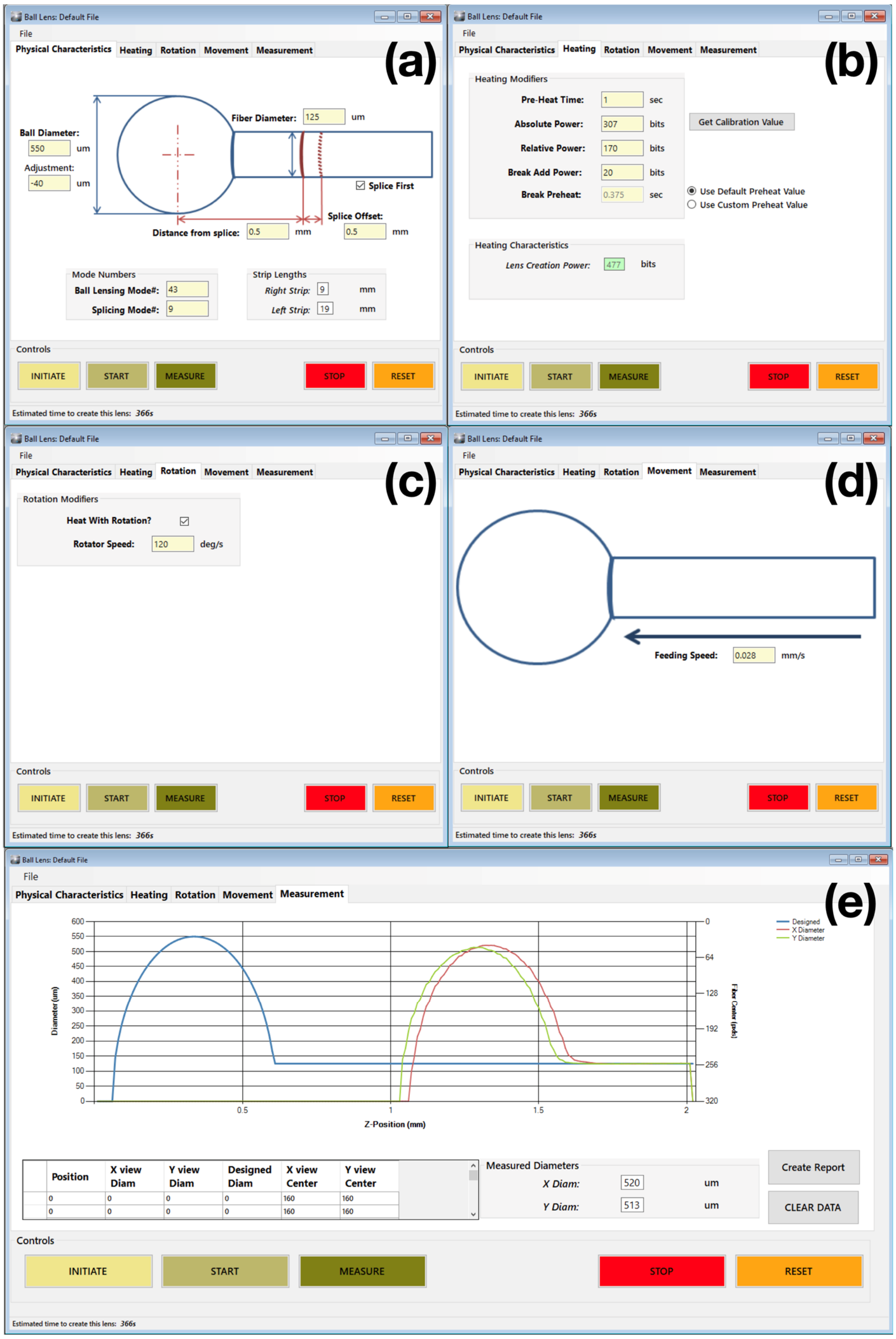

Figure S2. Settings of ball resonators manufacture: (a) Phisycal characteristics settings; (b) Heating settings; (c) Rotation settings; (d) Movement settings; (e) Measurements of fabricated ball resonators.

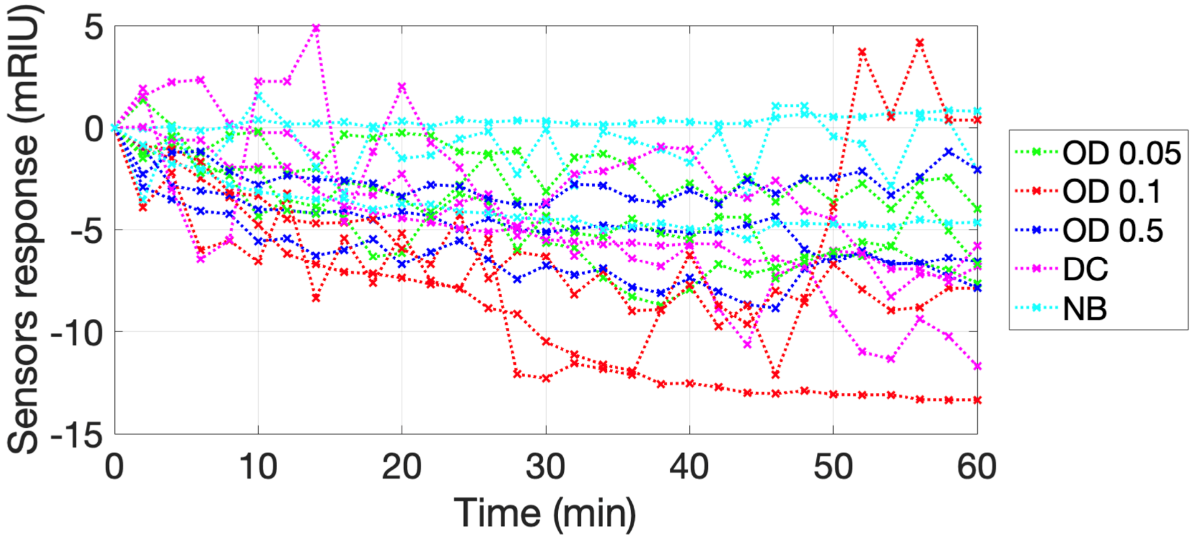

Figure S3. View of all response of all experiments over time.

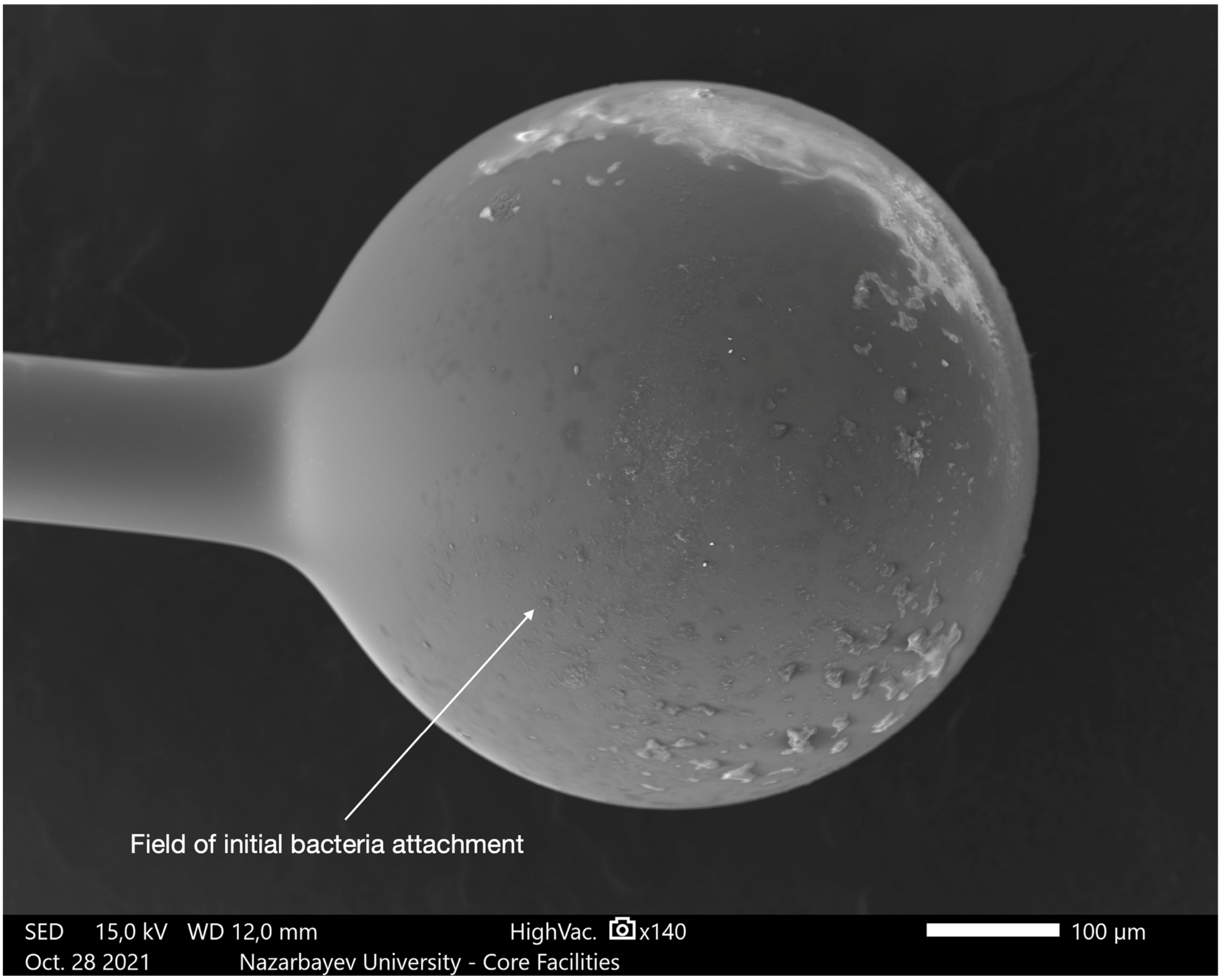

Figure S4. Scanning electron microscopy of OD=0.5 sample. The fealds of bacteria initial attachments are seen.

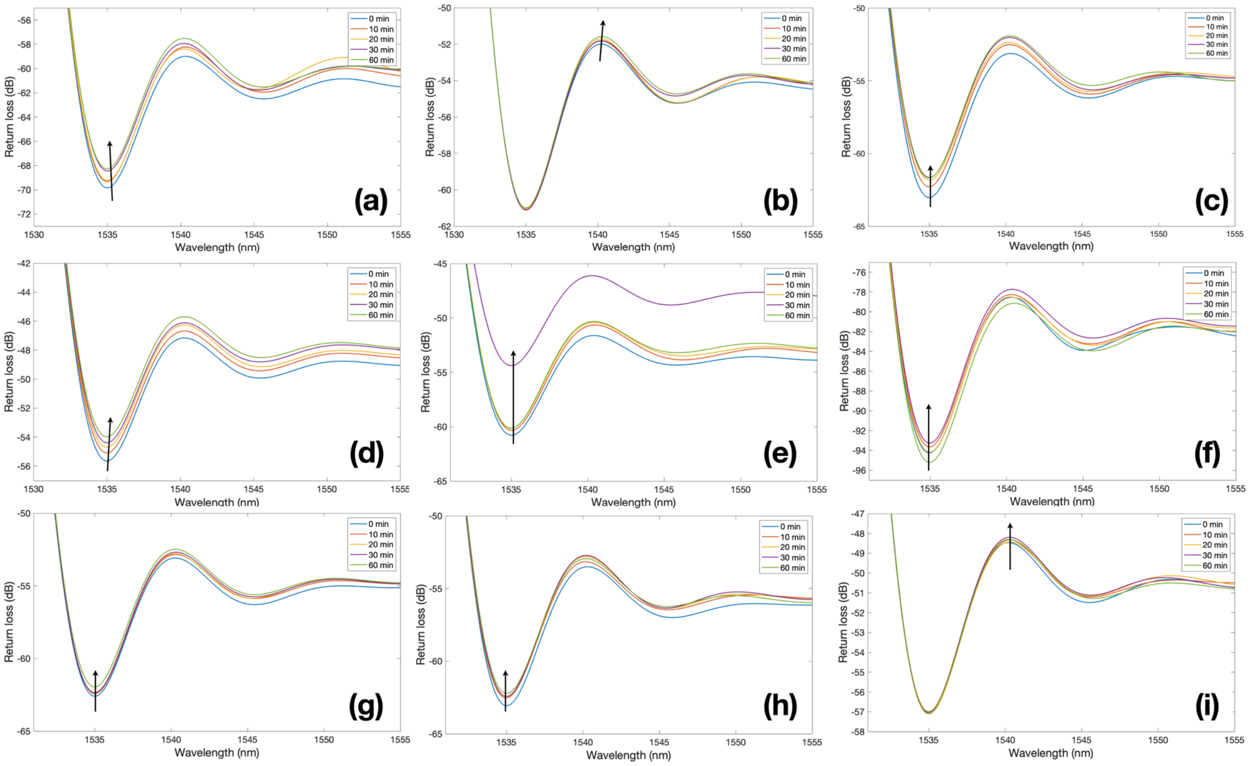

Figure S5. Spectra of ball resonators: (a)-(c) ball resonators used for OD=0.05 experiment with diameters 535-541μm, 541-534 μm, 526-521 μm respectively; (d)-(f) ball resonators used for OD=0.1 experiment with diameters 569-561 μm, 544-538 μm, 543-539 μm respectively; (g)-(i) ball resonators used for OD=0.05 experiment with diameters 532-523 μm, 523-521 μm, 520-513 μm respectively.

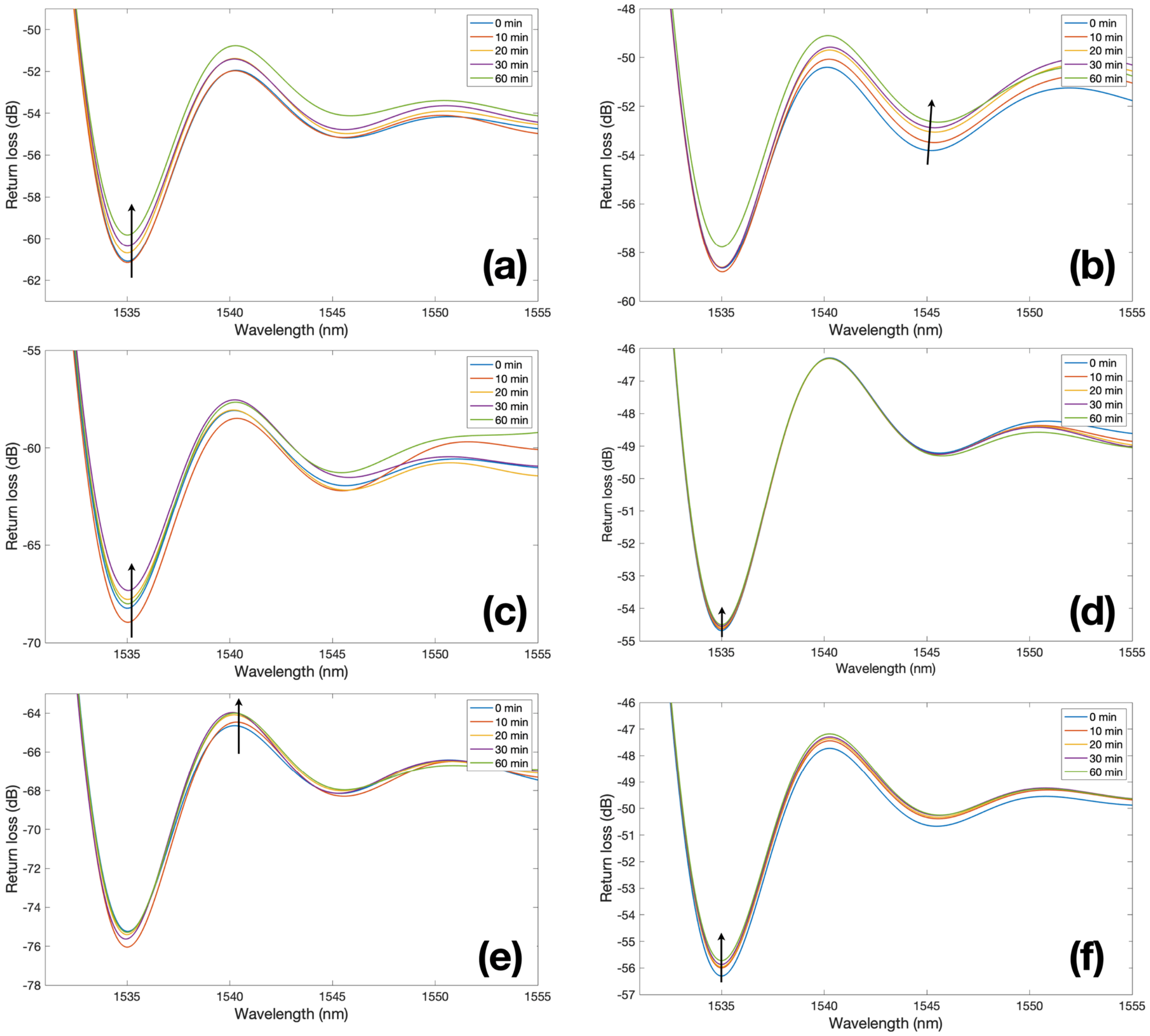

Figure S6. Spectrs of ball resonators used for controls: (a)-(c) ball resonators used for control DC with diameters 565-559 µm, 567-564 µm, 571-564 µm respectively; (d)-(f) ball resonators used for control NB with diameters 568-561 µm, 569-563 µm, 570-564 µm respectively.

Table S1. Size and sensitivity of all BRs used in experiments (NB – nutrient broth, DC – dead cells, bacteria OD=0.5, OD=0.1, OD=0.05).

#	Diametr (um)	Analyte	Sensitivity (dB/RIU)	R2	At the wavelength (nm)	Corresponding to the index	Experiment
1	573-565	NB	-90,59	1,00	1 552,83	9 364,55	NB
2	568-561	NB	-108,38	1,00	1 552,75	9 321,82	NB
3	569-563	NB	-92,26	0,95	1 573,42	21 463,19	NB
4	532-523	0,5	-104,46	0,97	1 552,98	9 453,06	DC
5	523-521	0,5	-96,41	0,98	1 571,60	20 407,15	DC
6	520-513	0,5	-98,66	0,95	1 553,14	9 553,78	DC
7	535-541	0,05	-127,52	0,98	1 552,85	9 379,81	OD=0.05
8	541-534	0,05	-128,37	0,98	1 552,55	9 202,78	OD=0.05
9	526-521	0,05	-122,32	0,97	1 580,55	25 577,46	OD=0.05
10	569-561	0,1	-106,03	0,99	1 552,88	9 398,12	OD=0.1
11	544-538	0,1	-144,28	0,99	1 565,42	16 799,54	OD=0.1
12	543-539	0,1	-101,64	0,95	1 570,99	20 050,05	OD=0.1
13	565-559(1)	DC	-90,71	1,00	1 552,91	9 413,38	OD=0.5
14	567-564	DC	-170,43	0,95	1 551,64	8 656,45	OD=0.5
15	571-564	DC	-115,24	0,99	1 582,89	26 923,45	OD=0.5
